# Work-life balance predicted work ability two years later: a cohort study of employees in the Swedish energy and water sector

**DOI:** 10.1186/s12889-021-11235-4

**Published:** 2021-06-24

**Authors:** Erik Berglund, Ingrid Anderzén, Åsa Andersén, Per Lindberg

**Affiliations:** 1 Department of Public Health and Caring Sciences, Uppsala University, Box 564, SE-751 22 Uppsala, Sweden; 2 Department of Occupational Health Science and Psychology, University of Gävle, Gävle, SE-801 76 Sweden

**Keywords:** Work-life balance, Work ability, Physical demands, Mental demands

## Abstract

**Background:**

Work-life balance (WLB) is the extent to which individual’s multiple life roles and demands carry over between each role. WLB can be divided into work interference with personal life (WIPL) and personal life interference with work (PLIW). This study aimed to investigate longitudinal associations between WIPL, PLIW and work ability outcomes.

**Methods:**

In this cohort study, 224 employees in the energy and water sector in Sweden were followed-up over 2 years. Three questions derived from the Work Ability Index were used for measuring work ability outcome: current work ability compared with lifetime best; work ability regarding physical; and mental demands. Logistic regression models were used to analyse longitudinal associations between work ability and WIPL and WIPL respectively, controlling for workplace (company), position at work, experience of leadership quality, demographics, and work ability.

**Results:**

Work ability compared to lifetime best were associated with WIPL in the adjusted logistic regression models (odds ratio (OR) 1.77, 95% confidence interval (CI) 1.15–2.73), and PLIW (OR 3.34, 95% CI 1.66–6.74). Work ability regarding physical demands was associated with WIPL (OR 1.60, 95% CI 1.07–2.40). Work ability regarding mental demands was associated with WIPL (OR 1.59, 95% CI 1.03–2.44) and PLIW (OR 2.88, 95% CI 1.31–6.32).

**Conclusion:**

In this two-year longitudinal study, lower WIPL predicted good/excellent overall work ability compared with lifetime best, higher work ability regarding physical and mental demands, and lower PLIW predicted good/excellent overall work ability compared with lifetime best and higher work ability regarding and mental demands.

## Background

Work ability is generally understood as the capability and capacity to be put into effective operation, among employees work ability concerns how well a worker is able to perform his or her work [1–3]. Even if there is no uniform definition of work ability in the literature [4]; work ability often includes physical, mental and social resources [5]. The concept of work ability may be divided into general and specific work ability [6]. Having work ability in a general sense is having the health, standard basic competence and the basic occupational virtues required for perform some kind of job, assuming that the work tasks are reasonable and that the work environment is acceptable. Having work ability in a specific sense, is having (some set of) the required manual, intellectual and social competence together with health and the relevant job-specific virtues that are required for managing the specific work tasks. Work ability has been shown to have predictive value for several outcomes, including; productivity [7], sick leave/absence [8, 9], job survival [10], work-related disability [11], and mortality [12].

Several factors have been proposed to influence employee’s work ability [5], e.g. individual factors, such as physical, psychological and cognitive functioning [13, 14]. There are also environmental factors of importance, which can be found both in the physical and social environment, and within or without the individuals’ workplace [13, 15]. Leadership constitute one dimension, “leadership climate”, of the psychosocial work environment that could have a prospective relationship with work ability [16, 17]. In the occupational health literature, the concept of work ability is often considered to be depending on the balance between personal resources and work demands [5], and models such as the Job Demands-Resources [18], and the Spillover model [19], are often applied. Factors outside the workplace has gained in interest, and especially the intersection between work life and private life [20], and scholars have produced a substantial body of theoretical literature on the linking of work and private life [20, 21]. Work-life balance (WLB) is a broad concept and much similar to the concept of work-family balance.

The concept of WLB is often drawn from individual’s multiple life roles and from the recognition that non-work demands may carry over into the work, and adversely influence the individual in several ways. Several different definitions have been proposed [22, 23]. A quite ordinary understanding is that WBL reflects an individual’s orientation across different life roles, and is an inter-role phenomenon [23–25]. Especially one theory in the occupational stress literature has particular relevance for guiding research on WLB, namely role theory [26]. Role theory is based on the premise that an individual’s life is comprised of several roles and the responsibilities that follow each role, including roles at work, as well as roles outside of work [27]. WLB can be fractionated into how much one role in life interferes with another role, such as if work interferes with personal life or vice versa. The theory states that there is a risk for a role conflict, or imbalance, to occur if pressures occur at the same time in two (or more) sets, especially if compliance with one set makes it more difficult to comply with the other set. Empirical research supports that work-life imbalance is associated with worse work related outcomes [28], and vice versa, a good WLB are considered to increase outcomes such as productivity [29], job and life satisfaction [30].

A workforce with long-term sustainable work ability is important to promote, not least because of the ageing population [31, 32]. Younger adults who are entering the work force today are expected to have a longer working life than preceding generation and therefore needs to maintain a sufficient work ability over a longer time frame [33, 34]. The work ability can be affected by a number of different factors and there is still a lack of a knowledge in the field; a not fully explored and interesting consideration is how WLB predicts work ability. The aim of this study was to investigate if lower interferes between work and private life predicted higher self-assessed work ability among white and blue collar workers in the energy and water sector in Sweden.

## Methods

### Setting and study population

**Fig. 1:**
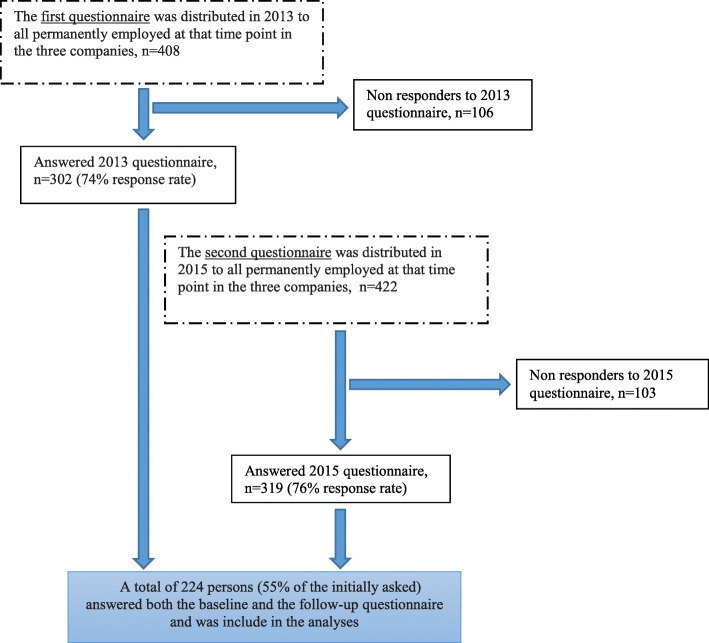
Flow-chart of the study population and the data collection process

This is a two-year longitudinal, questionnaire based study performed in three companies in the energy and water sector in Sweden. The present study is a part of an evaluation of the GodA-project (Swedish abbreviation for “Good work environment and healthy workplace”) [35]. GodA was inspired by the Practices for the Achievement of Total Health (PATH) model [36], and the intention was to carry out a PATH-based intervention aimed at enhancing internal communication in one of the companies (company 3), but the intervention was however not implemented to full extent due to organizational changes in the company at the time. In the present study, all employees in both the intervention and the control companies were treated as one cohort within a longitudinal study design, however the analyses were adjusted for company. The inclusion criteria were being permanently employed and in service in one of these companies at the time for the data collection. Both white and blue collar workers were included, as well as employees on short-term and/or part-time sick-leave and those who were home caring for a sick child. Employees on long-term sick-leave, parental leave or on a leave of absence were excluded. The questionnaires were distributed by e-mail, but there was also a paper-and-pencil questionnaire option for a few employees who did not feel comfortable using a computer. The baseline questionnaire, Time 0 (T_0_), was distributed in 2013 to all 408 employees in the three companies. A total of 302 employees answered the T_0_ questionnaire making a response rate of 74%. The follow-up questionnaire, Time 1 (T_1_), was disturbed in 2015 to all employed and in service at the time, 422 employees, of which 319 answered the questionnaire making a response rate of 76%. A total of 224 employees answered both the baseline and the follow-up questionnaires (55% of the initially asked) and form the study population in this study. See the flow-chart in Fig. 1. In addition, demographic data and information about work position were obtained from administration registers of the three participating companies.

### Explanatory measures

Data gathered in 2013 related to demographics, employment and WLB were used as explanatory measures in this study.

#### Demographics

Information about gender and age were obtained from the administration registers of the three participating companies. Educational level (categorized as compulsory school, secondary school or university) was collected through the questionnaires.

#### Employment

Data of employment was obtained from the administration registers of the three participating companies, which company the employees had their contracts, and which position the employees hold (categorized as production, administration and management position). Experienced leadership quality in the workplace was based on an index with seven indicators that was developed for this study. The following statements were used for measuring leadership quality: “Our managers treat us employees in a fair way”, “Our managers are hopeful even at setbacks”, “The information from the managers is clear to me”, “My immediate superior is easy to reach”, “My immediate superior is committed to what I do”, “My immediate superior is responsive if I have ideas and opinions”, and “My immediate superior contributes when problems needs to be quickly solved”. Answers were collected with the following alternatives: 1. Not at all; 2. To a small extent; 3. Partially; 4. Highly; 5. Very much; and 6. Extremely high. Experience of leadership quality were calculated into an index ranging from 1 to 6, were a higher score indicated better experience of leadership quality.

#### WLB

In research, the concept of WLB can be measured in several ways [22, 37, 38]. In this study, the measure for WLB was based on six items from the Fisher, Bulger and Smith’s WLB scale from 2009 [39]. The original scale assesses four dimensions: work interference with personal life (WIPL) and personal life interference with work (PLIW), work enhancement of personal life, and personal life enhancement of work. In this study, three questions were based of the WIPL dimension, and three items were based of the PLIW dimension. Both WIPL and PLIW were measured as how much each of these two dimensions affect (or interfere) on the other dimension. WIPL was assessed with answers to the following three statements: “When I come home from work I am too tired to do the things I would have liked to do”, “My private life doesn’t look the way I would like it to be because of my work”, and “I often overlook personal needs because of demands in my work”. PLIW was assessed with answers to the following three statements: “My work suffers from what’s happening in my private life”, “I would like to devote more time to work if it wasn’t for everything that happens in private life”, and “I’m too tired to be effective at work because of everything that happens in my private life”. Answers regarding WIPL and PLIW were collected with the following alternatives: 1. Almost all the time; 2. Often; 3. Sometimes; 4. Rarely; and 5. Not at all. WIPL and PLIW were both calculated into indices, ranging from 1 to 5. Higher scores indicated a lower interference and were interpreted as a more advantageous work-life balance, this direction of coding the answers has previously been used for the measure [40]. The WIPL and PLIW were used as separate factors in the analyses.

### Outcome measures

The outcome, self-assessed work ability, was measured at baseline and at follow-up. The first three questions, out of the original seven, from the Work Ability Index (WAI) were used in this study [41]. It is common to use parts of the WAI and not all questions due to space considerations in the questionnaire and high correlations among the indicators and the total score [42, 43]. The first question in WAI is known as the Work Ability Score (WAS) and asses current work ability compared with lifetime best [44], the question is outlined: “Assume that your work ability at its best has a value of 10 points. How many points would you give your current work ability?” This question is scored from 0 to 10. In this study, a cut-off score of 7 was used to dichotomized WAS into: Poor/moderate work ability (score 0–7) and good/excellent work ability (score 8–10) compared to lifetime best, for the binary logistic regression analyses. The cut-off score was chosen due to previous research which often classify a score of 0–7 points as poor and moderate, and 8–10 points as good and excellent work ability in WAS [45, 46]. The second WAI question concerned work ability regarding physical demands: “How do you rate your current work ability with respect to the physical demands of your work?” The third WAI question concerned work ability regarding mental demands: “How do you rate your current work ability with respect to the mental demands of your work?” The second and third WAI questions were assigned with five response options: 1. Very poor; 2. Rather poor; 3. Neither good nor poor; 4. Rather good; and 5.Very good. In this study, the work ability concerning physical and mental demands was dichotomized with a cut-off score of 4: into lower work ability (=0) and higher work ability (=1), for the binary logistic regression analyses. The three dimensions of WAI were analyzed separately.

### Statistical analysis

The data were analyzed using Chi-square tests to compare differences in percentage distributions and ANOVA for test differences between mean values. Spearman’s rho was used to explore the bivariate relationships between variables and used to summarize the data. Binary logistic regression analyses were used to study the longitudinal associations between the predictors at T_0_ and the outcomes at T_1_. A stepwise approach was performed for the logistic multiple regression models using sets of independent variables. Model 1 included PLIW and WIPL at T_0_. Model 2 included Model 1, company, position at work and experience of leadership quality at T_0_. Model 3 included Model 2 and demographics at T_0_. Model 4 included Model 3 and the baseline work ability measure as was used as outcome in the current analysis. When work ability compared with lifetime best at T_1_ was the dependent variable, work ability compared with lifetime best at T_0_ was controlled for, and when work ability regarding physical demands at T_1_ was the dependent variable, work ability regarding physical demands at T_0_ was controlled for, and so on. Adjusting for baseline work ability reduces the risk for “reverse” effects as a plausible explanation for relationships between predictors and outcomes [16]. All tests were two-sided, and a *p* value ≤0.05 was considered statistically significant. Statistical Package for the Social Sciences (SPSS)® version 25 (IBM SPSS Statistics for Windows, Armonk, NY: IBM Corp Chicago, IL, U.S.A.) was used for all statistical analyses.

## Results

The study population consisted of 73% men and 27% women and the average age of the study population was 47 years. Secondary or equivalent school was the most common completed education level. Among the study participants, 32% worked in production, 50% in administration, and 18% had a management position. The distribution of demographics in the study population are shown in Table 1.

**Table 1:** Baseline demographics and characteristics of the study population, distributed by company

		Company 1 ***n*** = 30 (13.4)	Company 2 ***n*** = 108 (48.2)	Company 3 ***n*** = 86 (38.4)	Total ***n*** = 224 (100)
Sex, n (%)	Male	20 (66.7)	81 (75.0)	63 (73.3)	164 (73.2)
	Female	10 (33.3)	27 (25.0)	23 (26.7)	60 (26.8)
Age	Md, Mean (±SD)	48, 47.1 (10.4)	47, 47.3 (9.2)	48, 46.6 (10.0)	48, 47.0 (9.6)
Education, n (%)	Primary school	8 (26.7)*	11 (10.2)*	22 (25.6)*	41 (18.3)
	Secondary school or equal	10 (33.3)*	67 (62.0)*	43 (50.0)*	120 (53.6)
	University	12 (40.0)*	30 (27.8)*	21 (24.4)*	63 (28.1)
Position at work, n (%)	Production	9 (31)	29 (26.9)	34 (39.5)	72 (32.4)
	Administration	14 (48.3)	62 (57.4)	35 (40.7)	111 (49.8)
	Manager	6 (20.7)	17 (15.7)	17 (19.8)	40 (17.9)
Experience of leadership quality^a^	Md, Mean (±SD)	4.0, 4.0 (0.8)	4.0, 3.9 (0.9)	4.0, 3.9 (0.9)	4.0, 3.9 (0.9)
Work-life balance	WIPL, Mean (±SD)^b^	3.49 (1.1)	3.79 (0.9)	3.76 (0.9)	3.74 (0.9)
	PLIW, Mean (±SD)^b^	4.67 (0.5)	4.58 (0.5)	4.58 (0.6)	4.59 (0.5)

Figures as number (n) or percentages if not stated otherwise. Pearson Chi-Square test was used for distributions and ANOVA test was used for mean

^a^Experience with leadership quality was measured with an index, ranging from 1 to 6. Higher score indicates better experience with management

^b^Work interference with personal life (WIPL) and personal life interference with work (PLIW), was both three item index scales, ranging from 1 to 5

*P ≤ 0.05

### Work-life balance

On average WIPL score was 3.7 (SD = 0.9) and PLIW score was 4.6 (SD = 0.5), in the total study population at baseline. No statistically significant differences were found between the three company’s employees regarding WIPL and PLIW, see Table 1.

### Work ability among the employees

In the total study sample, a majority of the employees (75%) reported good/excellent work ability compared to lifetime best, and 25% reported a poor/moderate work ability compared to lifetime best, at follow-up. When the 4-point cut-off of was used 59% reported a higher work ability regarding physical demands and 41% reported a higher work ability regarding mental demands at follow-up, see Table 2. There were no statistically significant differences between those workers who answered at baseline but not at follow-up and those who responded to both questionnaires regarding baseline WAS, work ability regarding physical and mental demands.

**Table 2:** Work ability measures at baseline and follow-up

		Baseline (T_**0**_)	Follow-up (T_**1**_)
Work ability compared to lifetime best	Md, Mean (±SD)^a^	7.0, 6.5 (1.4)	9.0, 8.3 (1.5)
	Poor/moderate work ability compared to lifetime best^b^	73.7	25.1
	Good/excellent work ability compared to lifetime best^b^	26.3	74.9
Work ability regarding physical demands	Md, Mean (±SD)^c^	5.0, 4.4 (0.7)	5.0, 4.5 (0.6)
	Lower work ability regarding physical demands^d^	46.0	41.3
	Higher work ability regarding physical demands^d^	54.0	58.7
Work ability regarding mental demands	Md, Mean (±SD)^c^	4.0, 4.2 (0.8)	4.0, 4.2 (0.8)
	Lower work ability regarding mental demands^d^	65.6	59.2
	Higher work ability regarding mental demands^d^	34.4	40.8

Figures are percentage if not stated otherwise

^a^Work ability compared with lifetime best was ranging from 0 (= worse) to 10 (= best)

^b^Work ability compared with lifetime best was dichotomized into: Poor/moderate work ability compared to lifetime best (=0) and good/excellent work ability compared to lifetime best (=1)

^c^Work ability regarding physical and mental demands was ranging from 0 (= worse) and 5 (= best)

^d^Work ability regarding physical and mental demands was dichotomized into: lower work ability (=0) and higher work ability (=1)

### Correlation matrix and logistic regressions

WIPL and PLIW both correlated with work ability compared to lifetime best and work ability regarding physical, as well as, mental demands, see Table 3.

**Table 3:** Correlation matrix among variables

	Min - max	1	2	3	4	5	6	7	8	9	10	11	12	13
1. Sex	1–2													
2. Age	21–64	-,09												
3. Education level	1–3	.26**	−.25**											
4. Company	1–3	−.02	−.02	−.11										
5. Position at work	1–3	.14*	−.01	.38**	−.07									
6. Experience with leadership quality^a^	1–6	.06	.04	.07	.03	.19**								
7. WIPL^b^	1–5	−.20**	.07	−.27**	.04	−.24**	−.02							
8. PLIW^b^	1–5	.00	.14*	−.23**	−.024	−.11	−.02	.39**						
9. Work ability compared with the lifetime best at T_0_	0–10	−.03	−.10	−.10	−.14*	−.08	.06	.38**	.40**					
10. Work ability regarding physical demands at T_0_	1–5	−.13*	−.07	.10	.10	.14*	.19**	.24**	.14*	.46**				
11. Work ability regarding mental demands at T_0_	1–5	−.09	−.05	−.06	.046	−.06	.20**	.36**	.27**	.68**	.59**			
12. Work ability compared with the lifetime best at T_1_	0–10	−.02	−.07	−.02	.03	.02	.09	.34**	.37**	.56**	.38**	.48**		
13. Work ability regarding physical demands at T_1_	1–5	.08	−.09	.17*	.03	.16*	.22**	.22**	.17*	.33**	.44**	.36**	.53**	
14. Work ability regarding mental demands at T_1_	1–5	−.10	−.01	.03	−.05	−.02	.14*	.36**	.28**	.45**	.32**	.46**	.65**	.56**

The matrix has been calculated with Spearman’s correlation coefficient

Maximum (max), minimum (min) of the scale or index, and correlations indicated

^a^Experience with leadership quality was measured with an 7 item index ranging from 1 to 6

^b^Work interference with personal life (WIPL) and personal life interference with work (PLIW), was both three item index scales, ranging from 1 to 5

^c^Work ability outcome in the matrix was measured at follow-up

* Correlation is significant at the 0.05 level

** Correlation is significant at the 0.01 level

Work ability compared to lifetime best at follow-up, WIPL, PLIW, company, position at work, experience of leadership quality, demographics and baseline work ability compared to lifetime best were analyzed using binary logistic regression, see Table 4. There was a statistically significant association between WIPL and good/excellent work ability compared to lifetime best, in the adjusted logistic regression models (odds ratio (OR) 1.77, 95% confidence interval (CI) 1.15–2.73). There was also a statistical significant association between PLIW and good/excellent work ability compared to lifetime best (adjusted OR 3.34, 95% CI 1.66–6.74).

**Table 4:** Results of logistic regression models of factors predicting good/excellent work ability compared with the lifetime best

		Crude OR 95% CI	Model 1 OR 95% CI	Model 2 OR 95% CI	Model 3 OR 95% CI	Model 4 OR 95% CI
**Work-life balance**	WIPL^a^	2.12** (1.49 to 2.98)	1.72** (1.18 to 2.49)	1.90** (1.27 to 2.84)	1.93** (1.26 to 2.94)	1.77** (1.15 to 2.73)
	PLIW^a^	4.67** (2.59 to 8.43)	3.70** (2.01 to 6.80)	3.82** (2.01 to 7.26)	4.13** (2.08 to 8.21)	3.34** (1.66 to 6.74)
**Employment**	Company					
	Company 1	1		1	1	1
	Company 2	1.18 (0.45 to 3.09)		1.39 (0.46 to 4.25)	1.65 (0.52 to 5.25)	1.87 (0.58 to 6.08)
	Company 3	0.67 (0.25 to 1.74)		0.60 (0.20 to 1.81)	0.61 (0.20 to 1.88)	0.65 (0.21 to 2.07)
	Position at work					
	Production	1		1	1	1
	Administration	1.23 (0.68 to 2.22)		1.04 (0.46 to 2.36)	1.10 (0.45 to 2.67)	1.12 (0.46 to 2.73)
	Manager	1.77 (0.81 to 3.88)		2.85 (0.90 to 9.03)	3.41 (0.99 to 11.70)	3.55* (1.02 to 12.36)
	Experience of leadership quality^b^	1.37 (0.96 to 1.94)		1.45 (0.95 to 2.23)	1.45 (0.94 to 2.23)	1.48 (0.94 to 2.35)
**Demographic**	Gender					
	Male	1			1	1
	Female	0.81 (0.47 to 1.42)			0.97 (0.41 to 2.30)	1.01 (0.42 to 2.42)
	Age	0.96 (0.96 to 1.03)			0.97 (0.94 to 1.01)	0.98 (0.94 to 1.02)
	Education level					
	University	1			1	1
	Secondary school or equal	0.64 (0.29 to 1.43)			0.48 (0.16 to 1.43)	0.43 (0.14 to 1.33)
	Compulsory school	0.55 (0.24 to 1.28)			0.56 (0.15 to 1.20)	0.48 (0.13 to 1.79)
**Baseline work ability regarding work ability compared with the lifetime best**	Poor/moderate at T_0_	1				1
	Good/excellent at T_0_	8.91** (2.67 to 29.79)				4.63* (1.28 to 16.76)
Nagelkerke R Square (R^2^)			24%	31%	32%	36%
ΔR^2^				7%	1%	4%

Odds ratio (OR), 95% CI: 95% confidence interval of factors predicting good/excellent work ability compared with the lifetime best. Work ability compared with the lifetime best was dichotomized into: Poor/moderate work ability compared to lifetime best (=0) and good/excellent work ability compared to lifetime best (=1). *P ≤ 0.05, **P ≤ 0.01 ^a^Work interference with personal life (WIPL) and personal life interference with work (PLIW), was both three item index scales, ranging from 1 to 5 ^b^Experience with leadership quality was measured with an index, ranging from 1 to 6. Model 1 = PLIW+WIPL, Model 2 = Model 1 + company+position at work+experience of leadership quality, Model 3 = Model 2 + gender+age + education level, Model 4 = Model 3 + baseline work ability compared with the lifetime best

Work ability regarding physical demands at follow-up, WIPL, PLIW, company, position at work, experience of leadership quality, demographics and baseline work ability regarding physical demands were analyzed using binary logistic regression, see Table 5. There was a statistically significant association between WIPL and higher work ability regarding physical demands (adjusted OR 1.60, 95% CI 1.07–2.40). There was also a statistically significant association between experience of leadership quality and higher work ability regarding physical demands (adjusted OR 1.51, 95% CI 1.02–2.22).

**Table 5:** Results of logistic regression models of factors predicting higher work ability regarding physical demands

		Crude OR 95% CI	Model 1 OR 95% CI	Model 2 OR 95% CI	Model 3 OR 95% CI	Model 4 OR 95% CI
**Work-life balance**	WIPL^a^	1.49** (1.10 to 2.00)	1.41* (1.03 to 1.93)	1.62** (1.15 to 2.30)	1.84** (1.27 to 2.69)	1.60* (1.07 to 2.40)
	PLIW^a^	1.61 (0.98 to 2.65)	1.33 (0.78 to 2.26)	1.39 (0.79 to 2.45)	1.60 (0.86 to 2.98)	1.41 (0.72 to 2.76)
**Employment**	Company					
	Company 1	1		1	1	1
	Company 2	1.81 (0.99 to 3.30)		1.54 (0.64 to 3.72)	1.81 (0.72 to 4.57)	1.52 (0.55 to 4.17)
	Company 3	2.61* (1.15 to 5.92)		1.70 (0.69 to 4.20)	2.00 (0.78 to 5.12)	1.42 (0.51 to 3.98)
	Position at work					
	Production	1		1	1	1
	Administration	0.77 (0.40 to 1.47)		2.28* (1.17 to 4.46)	1.61 (0.77 to 3.39)	1.55 (0.69 to 3.49)
	Manager	1.15 (0.48 to 2.79)		3.31** (1.33 to 8.26)	2.49 (0.94 to 6.59)	1.91 (0.67 to 5.40)
	Experience of leadership quality^b^	1.68** (1.21 to 2.33)		1.59** (1.12 to 2.27)	1.62** (1.13 to 2.33)	1.51* (1.02 to 2.22)
**Demographic**	Gender					
	Male	1			1	1
	Female	1.53 (0.92 to 2.56)			1.36 (0.64 to 2.89)	1.95 (0.85 to 4.49)
	Age	0.99 (0.96 to 1.01)			0.98 (0.95 to 1.02)	0.99 (0.95 to 1.02)
	Education level					
	University	1			1	1
	Secondary school or equal	1.40 (0.76 to 2.58)			0.98 (0.42 to 2.30)	0.96 (0.38 to 2.44)
	Compulsory school	3.49** (1.72 to 7.08)			2.75 (0.92 to 8.25)	2.06 (0.63 to 6.76)
**Baseline work ability regarding physical demands**	Lower at T_0_	1				1
	Higher at T_0_	6.70** (3.70 to 12.14)				5.43** (2.78 to 10.62)
Nagelkerke R Square (R^2^)			5%	17%	22%	35%
ΔR^2^				12%	5%	13%

Odds ratio (OR), 95% CI: 95% confidence interval of factors predicting higher work ability regarding physical demands. Work ability regarding physical demands was dichotomized into lower work ability (=0) and higher work ability (=1) *P ≤ 0.05, **P ≤ 0.01 ^a^Work interference with personal life (WIPL) and personal life interference with work (PLIW), was both three item index scales, ranging from 1 to 5. ^b^Experience with leadership quality was measured with an index, ranging from 1 to 6. Model 1 = WIPL+PLIW, Model 2 = Model 1 + company+position at work+experience of leadership quality, Model 3 = Model 2 + gender+age + education level, Model 4 = Model 3 + baseline work ability regarding physical demands

Work ability regarding mental demands at follow-up, WIPL, PLIW, company, position at work, experience of leadership quality, demographics and baseline work ability mental demands were analyzed using binary logistic regression, see Table 6. There were statistically significant associations between, WIPL as well as PLIW, and higher work ability regarding mental demands, with adjusted OR 1.59, 95% CI 1.03–2.44, and adjusted OR 2.88, 95% CI 1.31–6.32, respectively.

**Table 6:** Results of logistic regression models of factors predicting higher work ability regarding mental demands

		Crude OR 95% CI	Model 1 OR 95% CI	Model 2 OR 95% CI	Model 3 OR 95% CI	Model 4 OR 95% CI
**Work-life balance**	WIPL^a^	2.05** (1.46 to 2.88)	1.76** (1.23 to 2.51)	1.78** (1.22 to 2.59)	1.92** (1.29 to 2.87)	1.59* (1.03 to 2.44)
	PLIW^a^	3.35** (1.78 to 6.34)	2.44** (1.27 to 4.68)	2.60** (1.33 to 5.11)	3.38** (1.64 to 6.96)	2.88** (1.31 to 6.32)
**Employment**	Company					
	Company 1	1		1	1	1
	Company 2	2.36* (1.12 to 4.90)		2.20 (0.84 to 5.74)	2.47 (0.91 to 6.71)	2.38 (0.83 to 6.83)
	Company 3	1.38 (0.64 to 2.97)		1.30 (0.49 to 3.48)	1.46 (0.54 to 3.99)	1.27 (0.44 to 3.68)
	Position at work					
	Production	1		1	1	1
	Administration	0.80 (0.44 to 1.47)		0.95 (0.48 to 1.87)	0.71 (0.32 to 1.56)	0.66 (0.28 to 1.56)
	Manager	0.83 (0.38 to 1.83)		1.02 (0.41 to 2.50)	0.71 (0.270 to 1.87)	0.80 (0.29 to 2.23)
	Experience of leadership quality^b^	1.46* (1.05 to 2.04)		1.58* (1.10 to 2.28)	1.65** (1.13 to 2.40)	1.49* (1.003 to 2.21)
**Demographic**	Gender					
	Male	1			1	1
	Female	0.86 (0.53 to 1.42)			0.72 (0.33 to 1.55)	0.68 (0.29 to 1.58)
	Age	1.00 (0.97 to 1.03)			0.99 (0.96 to 1.02)	1.00 (0.96 to 1.03)
	Education level					
	University	1			1	1
	Secondary school or equal	0.83 (0.45 to 1.54)			1.11 (0.44 to 2.77)	1.28 (0.46 to 3.53)
	Compulsory school	1.51 (0.78 to 2.95)			3.39* (1.06 to 10.82)	3.85* (1.07 to 13.86)
**Baseline work ability regarding mental demands**	Lower at T_0_	1				1
	Higher at T_0_	6.92** (3.74 to 12.78)				5.55** (2.75 to 11.20)
Nagelkerke R Square (R^2^)			16%	21%	26%	38%
ΔR^2^				5%	5%	12%

Odds ratio (OR), 95% CI: 95% confidence interval of factors predicting higher work ability regarding mental demands. Work ability regarding mental demands was dichotomized into lower work ability (=0) and higher

work ability (=1) *P ≤ 0.05, **P ≤ 0.01 ^a^Work interference with personal life (WIPL) and personal life interference with work (PLIW), was both three item index scales, ranging from 1 to 5. ^b^Experience with leadership quality was measured with an index, ranging from 1 to 6. Model 1 = PLIW+WIPL, Model 2 = Model 1 + company+position at work+experience of leadership quality, Model 3 = Model 2 + gender+age + education level, Model 4 = Model 3 + baseline work ability regarding mental demands

## Discussion

This study investigated longitudinal associations of WIPL, PLIW and work ability outcomes 2 years later among employees in the Swedish energy and water sector. This study found that WIPL predicted work ability compared to lifetime best as well as work ability regarding physical and mental demands. PLIW predicted work ability compared to lifetime best and work ability regarding mental demands.

WLB is experienced when demands from work are compatible with demands from other domains in life, e.g. family or leisure time, and a ‘balanced’ situation occurs when demands from one domain do not negatively affect activities in the other domains [38]. In this study, WLB was divided into two separate factors or dimensions, ‘work interference with personal life’ and ‘personal life interference with work’, and used as separate predictors. The main reason for this strategy was to explore what kind of “role-related” balance, work or private, in a person’s life that could have specific importance for the various work ability outcomes. The analyses showed that work ability regarding physical demands was influenced by WIPL, and work ability regarding mental demands was influenced by WIPL as well as by PLIW. The explanations behind these results needs further attention in the research. Possibly, physical work ability is more solely dependent on work conditions in general, while mental work ability is also sensitive for conditions outside the workplace and a person’s close community. Another, possible explanation for the results, is that the explanation might lie more in different types of jobs (and associated demands). To uphold a job that, to the largest part, have high physical requirements might be less dependent on the home conditions than on conditions at work. On the other side, a job that have high mental requirements seems to be conditioned also on home circumstances, possibly because thoughts and feelings do not stop influencing the employee’s mental state at the moment he or she leaves the home and enters the workplace.

The concept of WLB has gained in attention, and involved both governments, political institutions, companies employers and employees’ organizations in the discussions [38]. The concept of WLB has largely generated from role theory and the increasing interest in ‘work-life conflict’ as a topic of academic and practitioner debate [26, 47]. Work-life conflicts engenders thru the loss of resources (e.g. time) and the multiple roles that individuals occupy and the competing demands between work and home [48]. Work-life conflicts are also assumed to have increased in relevance for employees in recent years due to changes in demography, workplace, working hours, and communication technology enabling a closer contact with the workplace [47]. This has raised the interest for organizations to implement WLB policies and practices (a.k.a. organizational family-supportive or family-friendly policy’s). WLB practices usually refers to practices that includes support for dependent care, flexible work options, family or personal leave, flexible work hours (e.g., flextime, compressed work week, etc.), home-based work, job sharing, onsite childcare or assistance with childcare and eldercare services [47]. However, the purpose for implementing such practices may not always be to reduce work-life conflicts exclusively, other reason might e.g. be by offering these practices; organizations want to attract new employees as well as to retain existing ones [47, 49]. In the long run WLB practices often wishes to accomplish goals regarding increased organizational effectiveness and performance, which also have some empirical support [29, 49, 50].

The results of this study support the assumption that long-term work ability are impacted of WLB, and suggests that an approach managing WLB might influence work ability over time. Such interventions may be carried out in several ways, e.g. thru implementing concepts such as the “healthy workplace” concept [51]. In the concept of the health-promoting workplace it is stated that the individual also is responsible for his or her health behavior, and at the same time the individual’s health is also affected by factors in the environment which are beyond the individual’s control [52]. Interventions to promote health and work ability are therefore more likely to be effective if they are addressed both at individual and organizational level (e.g. environment).

In general, it is difficult to separate the work from the private life, thereby employers and supervisors needs to cooperate and share the responsibility to promote work ability resources [5]. Thru the use of e-technologies, much work have migrated into the homes and e-technologies have blurred the lines between work and home, and studies have showed inconsistent results regarding the relationship between work-home practices and WLB [53, 54]. In times with a pandemic, such as the current COVID-19 (Coronavirus) pandemic, more people are expected to work from home [55], which also may have significance for WLB and difficulties to separate work from private life.

This study showed that WLB influence work ability outcomes over time. Further investigations are needed regarding the long-term associations obtained in this study to determine potential causality between WLB and work ability. Further investigations need to focus more in detail on different sources for work ability, exploring which factors might be the most effective to influence in order to increase employees’ work ability. This study involved employees in the energy and water sector, nevertheless WLB is understood as general and probably predicts other employees work ability in a similar way. More research is needed to understand how WLB could be managed in different branches and contexts to promote long-term work ability, also in times when an increased proportion of work takes place from home, which may increase the risks for interference [55, 56]. However, data collection for this study was done in a branch and at a time when homework was less common.

### Strengths and limitations

The strengths of this study include the longitudinal approach, which makes it possible to study longitudinal association between predictors and outcomes. The response rate was reasonable according to what is expected in questionnaire-based follow-up studies.

This study also has some limitations worth noting. The data on work ability were self-reported, and therefore some of the respondents may have underestimated or overestimated their rate of work ability. No data on non-respondents were collected. To limit the impact of possible selection bias the model was adjusted for demographic variables such as gender, age and educational level.

Even if a fair degree of variance in the outcome measures was explained (Nagelkerke R Square ranging from 35 to 38%), there is still a lot of unexplained variance left in the outcome measures. This indicates that there are other factors, not assessed in the regression models that affect work ability among the employees. It can be discussed whether the directions of causality in the underlying assumption are appropriate. As this study is not randomized, potential confounders has not been distributed randomly between the groups, thus, differences in outcomes at the end of a study period cannot be attributed to the predictor factors with full certainty.

The healthy worker effect (HWE), is a commonly discussed form of selection bias in relation to occupational cohort studies [57, 58]. HWE occurs when improper choice of comparison groups is used in occupational studies. The main mechanisms behind the HWE in cohort studies are health-based selection of workers into employment, and/or health-based differential losses to follow-up (healthy worker survivor effect) [59, 60]. The risk for HWE in this study was lowered by the use of internal comparisons between employees [58], and by only using respondents from the baseline that were employed, and also answered the follow-up questionnaire. Another known bias is measurement reactivity or question–behavior effect, which are concepts linked to the broader ‘Hawthorne effect’ [61]. The question–behavior effect has been demonstrated for a variety of behaviors, and the theory states that it is a risk that research may stimulate new thinking and new behaviors on the phenomenon that the research focus on [62]. In this study, questionnaires were distributed to the employees, and some results from the baseline questionnaire were communicated back to the participating companies as feedback after T0. This feedback may have affected the companies and improved the outcomes, in general, at the follow-up. This may also have contributed to the general increase that occurs in the WAS scale during the study period.

## Conclusion

Work-life balance factors predicted good/excellent overall work ability compared with lifetime best, in this two-year cohort study. Lower levels of work interference with personal life predicted higher work ability regarding physical and mental demands, and lower levels of personal life interference with work predicted higher work ability regarding physical demands. This study suggests that better work-life balance may lead to higher work ability among employees in the energy and water sector.

## Data Availability

The datasets used and analysed during the current study are available from the corresponding author on reasonable request.

## Abbreviations

HWE: Healthy worker effect
WLB: Work-life balance
OR: Odds ratio
PATH: Practices for the achievement of total health
PLIW: Personal life interference with work
SPSS: Statistical package for the social sciences
WAI: Work ability index
WAS: Work ability score
WIPL: Work interference with personal life
